# Other PHAC publications

**DOI:** 10.24095/hpcdp.44.3.08

**Published:** 2024-03

**Researchers from the Public Health Agency of Canada also contribute to work published in other journals and books. Look for the following articles published in 2023:**

Gabet S, Thierry B, **Wasfi R**, et al. How is the COVID-19 pandemic impacting our life, mental health, and well-being? Design and preliminary findings of the pan-Canadian longitudinal COHESION study. BMC Public Health. 2023;23(1):2401. https://doi.org/10.1186/s12889-023-17297-w

John S, Joseph KS, Fahey J, **Liu S**, et al. Counterpoint: Are abnormal fetal growth indices valid predictors of neonatal morbidity and mortality? Paediatr Perinat Epidemiol. 2024;38(1):18-21. https://doi.org/10.1111/ppe.13025 

King EC, Zagrodney KAP, **Rabeenthira P**, et al. Why did home care personal support service volumes drop during the COVID-19 pandemic? The contributions of client choice and personal support worker availability. Health Services Insights. 2023;16: 11786329231210692. https://doi.org/10.1177/11786329231210692

**O’Sullivan B,** Zhong A, Yin LL, et al. The future of global health: restructuring governance through inclusive youth leadership. BMJ Global Health. 2023;8(11):e013653. https://doi.org/10.1136/bmjgh-2023-013653

**Prince SA, Lang JJ, de Groh M, […] Butler GP, […] Geneau R**. Prioritizing a research agenda on built environments and physical activity: a twin panel Delphi consensus process with researchers and knowledge users. Int J Behav Nutr Phys Act. 2023;20(1):144. https://doi.org/10.1186/s12966-023-01533-y 

Saragosa M, Zagrodney KAP, **Rabeenthira P**, et al. How might we have known? Using administrative data to predict 30-day hospital readmission in clients receiving home care services from 2018 to 2021. Health Services Insights. 2023;16:11786329231211774. https://doi.org/10.1177/11786329231211774

Souleymanov R, Akinyele-Akanbi B, Njeze C, […] **Kim J**, et al. Migration and health study: a socio-ecological analysis of sexual health among migrants in Manitoba, Canada. BMC Public Health. 2023;23(1):2438. https://doi.org/10.1186/s12889-023-17379-9

Ziam S, Lanoue S, McSween-Cadieux E, […] **Jean E**, et al. Validation of a framework for evaluating knowledge mobilization strategies: a Delphi method approach. Proceedings of the European Conference on Knowledge Management, ECKM. 2023;24(2):1510-5. https://doi.org/10.34190/eckm.24.2.1642

